# Bibliometric mapping of glioma classification research through main path, key route, and K-core analyses

**DOI:** 10.18632/oncotarget.28851

**Published:** 2026-03-31

**Authors:** Kayode Ahmed, Juan E. Núñez-Ríos

**Affiliations:** ^1^The University of Texas MD Anderson Cancer Center, Houston, TX 77030, USA; ^2^Universidad Panamericana, Facultad de Ciencias Económicas y Empresariales, Zapopan, Jalisco 45010, México

**Keywords:** glioma research, social network analysis, socio-clinical domains, web of science, networks

## Abstract

Burgeoning technological and clinical advances have significantly reshaped glioma classification. To assess the evolution of these changes, we analyzed bibliometric data from Web of Science to explore patterns in the socio-clinical domains of glioma classification research. Using network analysis, we built a direct citation network linking articles to authors, focusing on citations. Main Path Analysis provided an overview of research evolution, Key Route Analysis identified influential papers, and K-core analysis revealed densely connected articles. The network comprised 46,204 nodes and 231,432 arcs, highlighting DNA methylation profiling’s role in advancing molecular biomarker-based classification models. KRA emphasized advanced imaging and molecular techniques as key drivers, while K-core analysis identified articles cited at least 19 times. The findings indicate that the subset of articles focusing on glioma classification that incorporate social factors is relatively scarce in the analyzed data, in contrast to the prominence of epigenetic and imaging factors in the literature. Unlike previous studies that focused primarily on metrics such as the h-index, our approach identifies the limited but notable mention of social factors in glioma classification research, thereby highlighting a thematic gap. Through quantitative network analysis complemented by narrative interpretation, we uncovered patterns and substructures that offer deep insights into the evolving research landscape.

## INTRODUCTION

Gliomas are the most common intracranial tumors, accounting for approximately 32% of all central nervous system (CNS) tumors and 81% of malignant brain tumors in the United States [1]. Classifying gliomas is crucial for identifying distinct biological or pathological traits that aid the selection of appropriate treatments, optimize therapeutic strategies, and improve prognostic outcomes [2].

The current World Health Organization (WHO) classification of gliomas is based on histopathological features and molecular markers [3]. While understanding the biological and genetic aspects of gliomas are crucial for diagnosis and treatment, social factors also play an important role in patient outcomes. Factors like socioeconomic status, social isolation, and education can significantly affect the course of the disease. By considering these social elements in the socio-clinical domain of glioma research and classification, healthcare providers can better address the challenges of glioma diagnosis and management, leading to improved care and support for patients [4, 5].

Despite the relevance of social factors as risk factors contributing to the development of oncological diseases, their integration into glioma classification systems has received considerably less attention than traditional histopathological and molecular approaches [6, 7]. This omission possibly limits the ability to develop more holistic classification models that reflect not only the biological characteristics of the tumor, but also the social conditions that shape access to diagnosis, therapeutic adherence, and clinical outcomes. As a result, the link between the social context and the evolution of gliomas remains a black box for clinicians and researchers; hence, the classification of gliomas should move beyond the reductionist view that limits it to a purely biomedical issue, overlooking the systemic complexity of the disease and the contributory role of social factors.

With approximately six thousand articles published on glioma in 2023 [8, 9], glioma classification and prognosis are extensively researched. However, the large number of publications makes it challenging to keep up with clinical and social developments in the field. Bibliometric analysis (BA) effectively manages large data sets and improves understanding of the knowledge generated, mainly using computational methods [10]. In the context of glioma research, [11] and [12] indicated that BA often focuses on analyzing collaborations between countries, institutions, or research groups, without contextual emphasis on the social aspects of the disease. [6, 7]. Hence, the primary purpose of this study is to map the evolution of glioma classification research, emphasizing the integration (or lack thereof) of clinical and social factors. To this end, we conducted a bibliometric analysis using data from the Web of Science (WoS) database, specifically, a network of direct citations (elaborated with the cited references (CR) field of the WoS records) to identify the intellectual structure and relational dynamics that have shaped this field of study [13]. To analyze the structure of the aforementioned network, the following network techniques were used: (1) Main Path Analysis (MPA) to identify the central evolutionary trajectory in the network, seeking to identify the sequence of key articles that have defined the development of the field; (2) Key Route Analysis (KRA) to complement the analysis and reveal multiple critical routes or alternative trajectories that connect approaches, techniques and scientific communities, thus revealing interdisciplinary convergence; and (3) K-core analysis, to identify densely connected parts of the network, showing more consolidated thematic communities and highlighting the groups of articles that form the current methodological and conceptual basis of glioma classification. This combination of techniques provides a unique contribution by identifying key influential works and uncovering the structural properties of the knowledge network driving glioma classification research. Unlike previous bibliometric reviews that focus almost exclusively on quantitative impact metrics, our approach highlights the evolutionary logic of the field by identifying critical interdisciplinary bridges between clinical, molecular, and social dimensions. This enables us to propose an integrative framework for glioma classification, highlighting the need to incorporate social factors into diagnostic and prognostic models for a more comprehensive understanding of the disease.

Our systematic approach to the synthesis of this study is as follows: First, we reviewed the literature on glioma classification, examining both medical perspectives and other literature reviews. Second, in the methodology section, we detail our approach in analyzing bibliometric data, including the use of MPA, KRA, and K-core, offering a comprehensive framework for professionals. Finally, we conclude with a brief discussion of the social factors implicated in glioma classification.

This study is intended for the multidisciplinary team involved in glioma research and management, including healthcare professionals, researchers, and academics- especially for those exploring advancements in glioma research and bibliometric network analysis.

## RELATED WORKS

This section presents studies that have examined the methods and characteristics of classifying gliomas using WoS as the primary source of information. This database was selected because it covers a wide range of topics, has a rigorous publication selection process, and provides various options for downloading data [14].

[15] and [16] classified bibliometric studies on gliomas into two categories: non-systematic and systematic. Non-systematic contributions seek to review and evaluate concepts, methods, and theories to understand glioma classification techniques better. Under the bibliometric approach, systematic studies analyze and identify new areas of study and evaluate the development of techniques to improve pathological assessment and effective treatments. Both perspectives seek to bridge the theoretical and practical domains of neuro-oncology. Thus, studies on glioma and its classification seek to connect evidence-based approaches with management paradigms in neuro-oncology. [17] reported that prior works have focused on the evolution of molecular biology and imaging techniques to improve classification and drug design. Accordingly, [6, 18], and [19] agreed that studies focus on the following areas:

1. *Evolution in fundamental histological classifications*: [7] and suggest that efforts in this line of research focus on revising histological classifications of gliomas, focusing on their microscopic appearances and behaviors. This path has been crucial in distinguishing between different types of gliomas, such as astrocytomas, oligodendrogliomas, and glioblastomas, based on histopathological features [20]. However, the accuracy of the results remains a significant limitation because the subjective interpretation of glioma histopathological features affects the effectiveness of treatment decisions.
2. *Diagnostic history*: It is considered fundamental to study the classification of gliomas. It has evolved from autopsies and macroscopic inspections to integrating model molecular markers [21].
3. *Glioma epigenetics*: The main feature is that studying DNA methylation patterns has added complexity to classifying gliomas. The identification of the glioma CpG island methylator phenotype (G-CIMP) and its association with IDH mutations and prognosis has refined our understanding of glioma biology and gliomagenesis [22, 23].
4. *Use of biomarkers and advances in molecular studies*: Advances in biomarkers such as IDH1 and IDH2, TP53, TERT, and ATRX have allowed a more accurate classification of gliomas, improving diagnosis of the disease and prediction of the disease [24, 25].
5. *Histological studies*: Microscopic analysis of abnormal glial cells is important in this classification, as it provides vital information on cell size, genetic alterations, and tissue nature. These analyses allow the development of microlevel analyses of abnormal cells, which help to create predictive models to improve treatments [20, 26].
6. *Imaging studies*: This area highlights the collaboration between disciplines such as medicine, bioengineering, and informatics, which has developed sophisticated instruments to capture and analyze images. These studies have led to better disease detection and prognosis, as well as preoperative and postoperative segmentation of tumors for better genetic characterization and classification of gliomas [23, 27, 28].

The ideas mentioned above show that from a non-systematic perspective, glioma research focuses on specific areas, which has allowed advances in the understanding, technical aspects, and complex processes of glioma analysis. In contrast, the systematic or quantitative analysis of the bibliometric data has improved the understanding of this field [29]. [30] and [31] indicate that the literature review articles on glioma classification focus on several key areas:

1. *Advances or developments in research techniques*: In this aspect, [32] and [33] have explored collaboration between researchers, highlighting the incorporation of genetic and molecular features to monitor mutations and improve accuracy in lesion prediction. Collaborative research groups are vital in glioma classification, facilitating sharing information and resources to solve complex problems. [34] reported how researchers from different countries collaborated to standardize MRI diagnostic procedures and protocols during crises.
2. *Diagnosis and treatment*: In this area, we can mention the work of [35] and [36], who mapped collaborative networks, finding advances in MRI and computational simulations to generate high-definition images. They highlight that collaborative structures allow the formation of interdisciplinary networks that improve consensus and allow the exploration of aspects inaccessible to individual researchers. [26] highlighted how collaboration between geneticists, oncologists, bioinformaticians, and engineers improved the processing of electronic signals to detect gliomas.
3. *Histopathological classifications and biochemical advances*: [31] analyzed articles on glioblastoma, histology and biochemistry, using exploratory analysis and correlation techniques. They found that research on glioblastoma multiforme has stagnated due to lack of funding, although literature highlights its heterogeneity and dangerousness.
4. *Image classification, artificial intelligence, and robotics applications*: [37] performed meta-analyses to evaluate the efficacy of diagnostic and robotic surgical interventions supported by preoperative volumetric imaging. They concluded that robotic surgery is still exploratory and requires standardization of safety models, requiring interdisciplinary collaboration and increased funding [38]. They used VOSviewer to map collaborative relationships between institutions and analyzed common terms related to artificial intelligence and image classification techniques. [39] investigated the use of artificial intelligence to improve glioma diagnosis through image analysis by representing information using network visualization.

Most of the studies cited in this section employ BA to study the structure of the scientific community working on glioma-related topics rather than their classification. BA usually examines collaborations, citations, productivity, and predominant authorship. Some studies take a quantitative approach to analyze conceptual or thematic structure and often report descriptive statistics or discuss aspects such as innovation in diagnostic techniques. Table 1 includes some of the studies covered in our review and briefly outlines the search strategy with focus on understanding gliomas. Each article was individually reviewed by two authors in the creation of this table.

**Table 1:** Search strategy from articles reported in literature review

Study	Database	Search strategy	Search query	Analysis type	Goal
[40]	WoS Core Collection			Meta-analysis	Diagnosis and treatment
[30]	Medline	By topic	.mp. (antiangiogenic or anti-angiogenic).mp.	Meta-analysis	Treatment efficacy
[37]	Cochrane and PubMed	By concept or glioma characteristic	AND (biopsy OR biopsies) AND (robot OR robotic)	Correlation	Robotics application
[31]	Scopus	By terms	Glioblastoma Multiforme, GBM, Glioblastoma, and Grade IV Glioma	Correlation	Advances in glioma research
[21]	PubMed, WoS and Scopus	By keywords	glioma, ischemia, memory, aging, cognitive impairment, Alzheimer,	Manual examination	Advances in glioma research
[27]	PubMed	By keywords	“diffuse intrinsic pontine glioma”, “pontine glioma”, or “midline glioma”	Manual examination	Epigenetics
[18]	PubMed	By terms	”glioma”	Latent Dirichlet allocation	Topic identification
[41]	WoS Core Collection	By terms	“social cognition” OR “theory of mind” OR “mentaliz*» OR “empath*” OR “emotion recognition” OR “social problem solving” OR “social skills” OR “cognit*” OR “memory” OR “execut*» OR “attention” OR “information speed” OR “visual construction”) AND (“brain tumour*» OR “brain tumor*» OR “brain neoplasm*» OR “intracranial neoplasm*» OR “brain cancer*» OR “intracranial tumour*» OR “intracranial tumor*» OR “glioma*” OR “meningioma*» OR “primary central nervous system lymphoma*» OR “brain metastases” OR “brain etastasis” “social cognition” OR “theory of mind” OR “mentaliz*» OR “empath*» OR “emotion recognition” OR “social problem solving” OR “social skills” OR “cognit*” OR “memory” OR “execut*» OR “attention” OR “information speed” OR “visual construction”) AND (“meningioma*» OR “neurinoma*” OR “lowgrade glioma*” OR “low-grade glioma*” OR “primary central nervous system lymphoma*”	Manual examination	Topic identification
[38]	WoS	By topic	Title (Oncology* or neoplasms* or oncol-ogys*) AND (artificial intelligence* or machine learning* or deep learning* or neural network* or logistic regression* or random forest* or support vector machine* or fuzzy logic* or computer vision* automatic programming* or speech understanding* or autonomous robots* or intelligent tutoring* or intelligent agents* or neural network* or voice recognition* or text mining*. Publication Indexes= Science Citation Index Expanded (SCI-expanded), Social Sciences Citation Index (SSCI), Arts & Humanities Citation Index (A&HCI), Conference Proceedings Citation Index- Science (CPCI-S), Conference Proceedings Citation Index- Social Science & Humanities (CPCI-SSH), Emerging Sources Citation Index (ESCI), Current Chemical Reactions (CCR-Expanded), Index Chemicus (IC). Timespan=All years.	Descriptive statistics	Artificial intelligence and machine learning application
[35]	WoS Core Collection	By terms	TI=(an*esthesia or an*esthetic or narcotic or Propofol or etomidate or Opioid or *fentanyl or morphi* or Dexmedetomidine or midazolam or *caine or *flurane or ketamine or naltrexone or naloxone) AND TS=(tum*t or neoplasm or cancer or carcinoma) NOT TS=(non-cancer or “chronic pain”) AND TS=(prognos*s or outcome or recurrence or “overall survival” or “recurrence free survival” or “relapse-free survival” or proliferation or invasion or metastas*s) NOT TI=guideline or recommendation or consensus or “case report” or meta or review) AND Language=English	Relational analysis	The influence of anesthesia on tumor prognosis
[36]	Scopus	By terms	(intra-arterial) AND (therapy OR treatment) AND (brain tumor OR glioma)	Descriptive analysis	Therapies for brain tumors treatment
[6]	WoS Core Collection	By terms	“Oncolytic virus*» OR “Oncolytic virotherap*» ((brain OR “central nervous system” OR CNS OR intracranial) NEAR/1 (cancer* OR anticancer* OR tumor* OR tumour* OR oncology OR neoplasm* OR carcinoma* OR lymphoma*)) OR meningioma* OR astrocytoma* OR oligodendroglioma* OR oligoastrocytoma* OR glioma* OR glioblastoma* OR neuroblastoma* OR “leptomeningeal disease*» OR “leptomeningeal carcinomatosis*» OR “ependymoma*» OR “subependymoma*» OR “gangliocytoma*» OR “ganglioglioma*» OR “ganglioneuroma*» OR “ganglioneuroblastoma*» OR “chordoma*» OR “notochordal cell tumor*» OR “notochordal cell tumour*» OR “schwannoma*» OR “neurilemmoma*» OR “neurinoma*” OR “spinal cord tumor*» OR “spinal cord tumour*» OR “extradural tumor*» OR “extradural tumour*” OR “intradural extramedullary tumour*» OR “intradural extramedullary tumor*” OR “intradural intramedullary tumour*» OR “intradural intramedullary tumor*”	Correlation coefficient	Topic identification
[22]	WoS Core Collection	By topic	(“acute myeloid leukemia” OR “AML”) AND (“targeted therapy*”)	Statistical analysis	Topic prediction
[12]	WoS Core Collection	By terms	TI = “glioma stem cell*” OR TI = “glioblastoma stem cell*” OR TI = “GBM stem cell*” OR TI = “GBM stem-like cell*” OR TI = “brain tumor-initiating cell*” OR TI = (“cancer stem cell*” AND (“brain” OR “glioma ” OR “glioblastoma” OR “GBM”)) OR TI = “glioma progenitor cell*”	Correlation analysis	Topic prediction
[42]	WoS	By keyword	(“machine learning” OR “deep learning” OR “artificial intelligence” OR “radiomics”)	Descriptive and correlational analysis	Artificial intelligence and machine learning application
[39]	WoS Core Collection	By keyword	(“brain” or “cerebrum” or “encephalon” or “pericranium” or “cerebral*” or “central nervous system”	Correlation analysis	Advances or developments in research techniques

Regarding social factors, [43, 44], and [45] converge by exploring the relationship between symptoms and quality of life in patients with diffuse glioma. They mapped symptoms to identify associations between depression, cognition, and brain tumor-related symptoms, creating symptom networks and analyzing their correlations. Although they did not address social risk factors implicated in gliomagenesis, they demonstrated that SNA could unveil the multidimensionality of symptoms in glioma studies.

Our article focuses on the following questions: What disciplinary approaches impact glioma classification, what factors have been considered to broaden this classification, and what social factors are associated with glioma classification? Incorporating social factors is crucial because, from a genomics perspective, it would lead to a more accurate prediction of patient prognosis and survival outcomes compared to classifications based only on histology and traditional clinical parameters alone [2, 41]. Furthermore, it could lead to the efficient utilization of available resources for adequate patient care.

Thus, our BA differs from previous ones in that it seeks to utilize information on social factors in glioma classification, research, and management to foster awareness regarding the importance of integrating social factors into glioma research, improve screening and documentation of social factors by incorporating them into specialty guidelines, and reporting results in a stratified manner. Incorporating social factors to enhance existing classification and diagnostic modalities that assess glioma risk can inform optimal personalized patient care practices. SNA allows for managing large amounts of heterogeneous data in databases such as WoS and unveiling relationships that traditional analytical methods do not capture [46, 47].

## METHODOLOGY

We developed a search strategy to identify relevant literature on glioma research through BA while ensuring methodological transparency. Filters, impact, time window, and specific inclusion and exclusion criteria used to pragmatically select articles that comprise the data are presented in Table 2. Boolean operators were applied to minimize ambiguity and accurately intersect key terms related to glioma and bibliometric techniques. The use of TS (Title, Abstract, Keywords) rather than ALL facilitated a focused yet comprehensive retrieval, minimizing irrelevant results while encompassing relevant studies. Terms such as “systematic review” and “meta-analysis” were incorporated to identify bibliometric reviews that synthesize research trends. The final query was iteratively refined to ensure its robustness and reproducibility in bibliometric research. Thus the adopted research query was: (TS=(“glioma” OR “brain tumor” OR “neuro-oncology” OR “cancer research” OR “glioblastoma” OR “astrocytoma” OR “oligodendroglioma”) AND TS=(“bibliometric analysis” OR “science mapping” OR “citation analysis” OR “co-word analysis” OR “co-citation” OR “systematic review” OR “meta-analysis”)) AND PY=(2015–2024) AND DT=(“Article” OR “Review”).

**Table 2:** Inclusion and exclusion criteria definition

Criteria	Description	Application
Type of literature	Studies for understanding and expanding glioma classification are explored or analyzed	Inclusion: Articles that: consider genetic, molecular, clinical factors, application of advanced techniques, patient care, or social aspects Exclusion: Grey literature, working papers, newsletters, editorial letters, treatments, or therapies without analyzing grading factors, individual case studies, drug application.
Type of source	The location or origin of the studies to be analyzed may be an aspect that allows an article to be included or rejected in the dataset.	Inclusion: Articles from journals or conferences. Exclusion: Book chapters.
Impact source	The reputation and impact of information sources is an aspect that must be verified to provide valuable data for analysis.	Inclusion: Q1 or Q2 sources and highly cited articles. Exclusion: Q3, Q4 sources.
Language	Studies that are available in English, given the prevalence of this language in the international scientific literature.	Exclusion: Studies published not in English.
Period	Our study aims to identify social factors that may impact glioma classification. Therefore, it is crucial to collect up-to-date data.	Inclusion: From 2018–2024. Exclusion: Studies prior to 2018.
Accessibility	It is important to be able to access both the database to retrieve the information and to access the articles.	Exclusion: Not accessible articles.
Relevance to research questions	Studies should be relevant in the context of the research questions posed as well as relate to the analytical perspective of interest.	Inclusion: Relevant to at least two research questions. Exclusion: Duplicated studies or studies that do not add new information to the existing classification criteria, omitting socio-genomic criteria.

Source: Adapted from [56].

Data collection, processing, and analysis followed the approach proposed by [48]. A brief description of the steps adopted is given below:

### Data acquisition

The bibliometric dataset was obtained from Web of Science (WoS) Core Collection, which aggregates peer-reviewed literature from multiple disciplines and provides standardized citation and reference metadata suitable for network analysis. We selected WoS for its rigorous indexing criteria, consistent citation format, and broad coverage of high-impact journals in neuro-oncology and related fields. We recognize that other databases can provide bibliometric data, such as Scopus, whose coverage usually overlaps or coincides with that of WoS. However, simultaneously integrating databases into a network-based study requires metadata curation and standardization processes that tend to increase the network’s structural noise without guaranteeing substantive changes to the identified paradigmatic core. In this sense, for the construction of networks, priority was given to the quality and homogeneity of the metadata offered by WoS [14] over absolute comprehensiveness of coverage.

The retrieved records included complete bibliographic information (title, authors, affiliations, abstract, keywords), cited references, and citation counts, enabling the construction of targeted citation networks and the subsequent application of main path analysis, key route analysis, and k-core decomposition. The selected time window (2015–2024) considers two primary aspects: first, the rapid updates of glioma classification frameworks following the incorporation of molecular biomarkers in the 2016 and 2021 WHO classifications [49, 50]; and second, the proliferation of studies based on computational approaches and multi-omic methodologies applied to the study of gliomas [51, 52].

The timepoint from 2015–2024 in WoS yielded 2,488 records according to the criteria defined in Table 2 and taking up the ideas of [53]. Given the significant changes in classification paradigms since 2018, we restricted our analysis to this period, aiming to capture methodologically and conceptually important contributions, as recommended by [54]. This timepoint (2018–2024) in WoS yielded 2,079 records which met criteria in Table 2 and was utilized for our analysis, indicating that 409 articles were excluded from our initial search result. The search string combined terms related to classification (such as “classification framework” and “subtype classification”) with terms specific to bibliometric analysis and systematic reviews, excluding from the outset clinical or therapeutic articles that do not explicitly address classification. Secondly, the results were filtered by document type (articles and reviews only), language (English), and source (WoS Core Collection only), which considerably reduced the initial universe. Thirdly, an impact filter was applied, considering only articles published in quartile one or quartile two journals and highly cited works. This approach is in line with the principle of cumulative advantage in citation networks, where a few highly influential works tend to concentrate a considerable proportion of citations and act as key nodes for the evolution of a field [55].

The configuration or combination of filters earlier described may introduce bias toward consolidated core results, which may be dominated by contributions oriented toward molecular, epigenetic, and imaging approaches. However, the design does not stray from, and is consistent with, the objective of our study, which seeks to shape the intellectual backbone of glioma classification rather than perform an exhaustive mapping of peripheral or emerging contributions. Hence, supplementing the study with other databases, such as Scopus or Google Scholar, would expand the network; however, the heterogeneity and noise of their metadata make it challenging to construct true acyclic networks. We also recognize that the strategy adopted could underrepresent recent articles in interdisciplinary journals or in gray literature, which is often disseminated beyond the dominant neuro-oncology channels.

**Figure 1:**
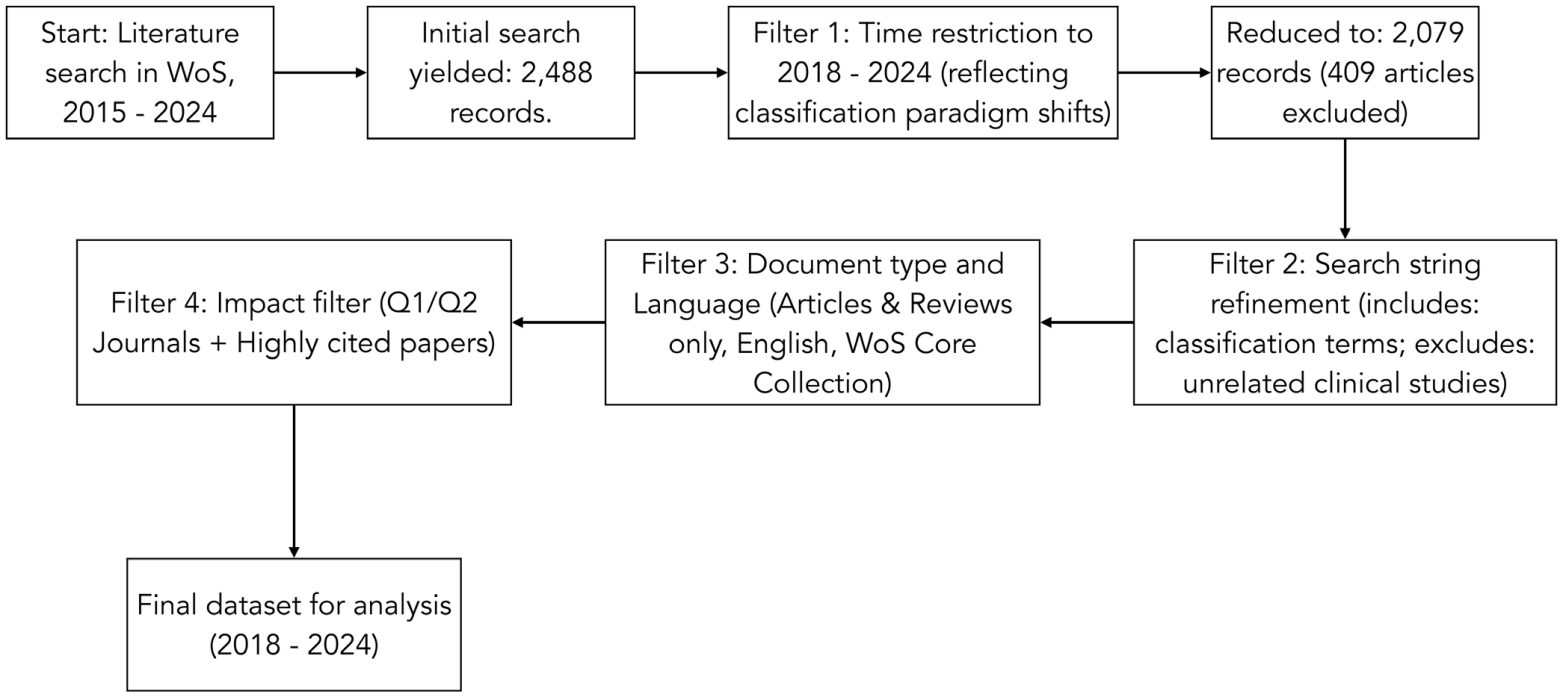
Search workflow. Source: Self-elaboration.

The textual workflow of our search strategy described above is succinctly depicted in Figure 1.

### Data cleaning and network elaboration

WoS2Pajek [57] was used to extract and organize the information in a network format. This tool allowed data cleaning and sorting, generating two two-mode networks: one for works and authors and another for works and keywords. This allowed us to get a first idea of the connections between these sets of nodes. Since these networks share a set of nodes (*works*), they can be transformed into a one-mode graph by multiplying networks, thus obtaining a network of keywords, journals, and co-authors.

### Analysis

Using SNA, we analyzed citations network (one-mode graph) [58]. We incorporated the ideas of [59] and [60] and used Pajek V64 6.01 [61] as an analytical tool. Subsequently, to carry out the analysis, we selected three specific techniques: *MPA, KRA, and K-core decomposition*, so that the integration of these techniques is aligned with the primary goal proposed for our work as these techniques have proven their robustness in analyzing directed networks [62, 63]. Although techniques such as co-citation clustering or topic modeling can support the detection of latent conceptual clusters [64], they were not selected because they do not preserve the temporal directionality of the relationships or citations, which is a limitation and makes them unsuitable for approaching questions or problems about directed networks or trajectories for the construction of knowledge or the identification of critical paths of development of a given field [65]. In contrast, MPA and KRA are used in directed networks, which not only help identify thematic groups, as co-citation analysis does, but also allows for evaluating the evolution of a field and highlighting key works that serve as pivots in that process [62]. On the other hand, since topic modeling is a natural language processing technique, it is usually applied to full texts [66], which limits the reconstruction of a directed network that allows us to observe scientific influence and theoretical-methodological dependencies between studies [67].

#### Degree distribution

**Figure 2:**
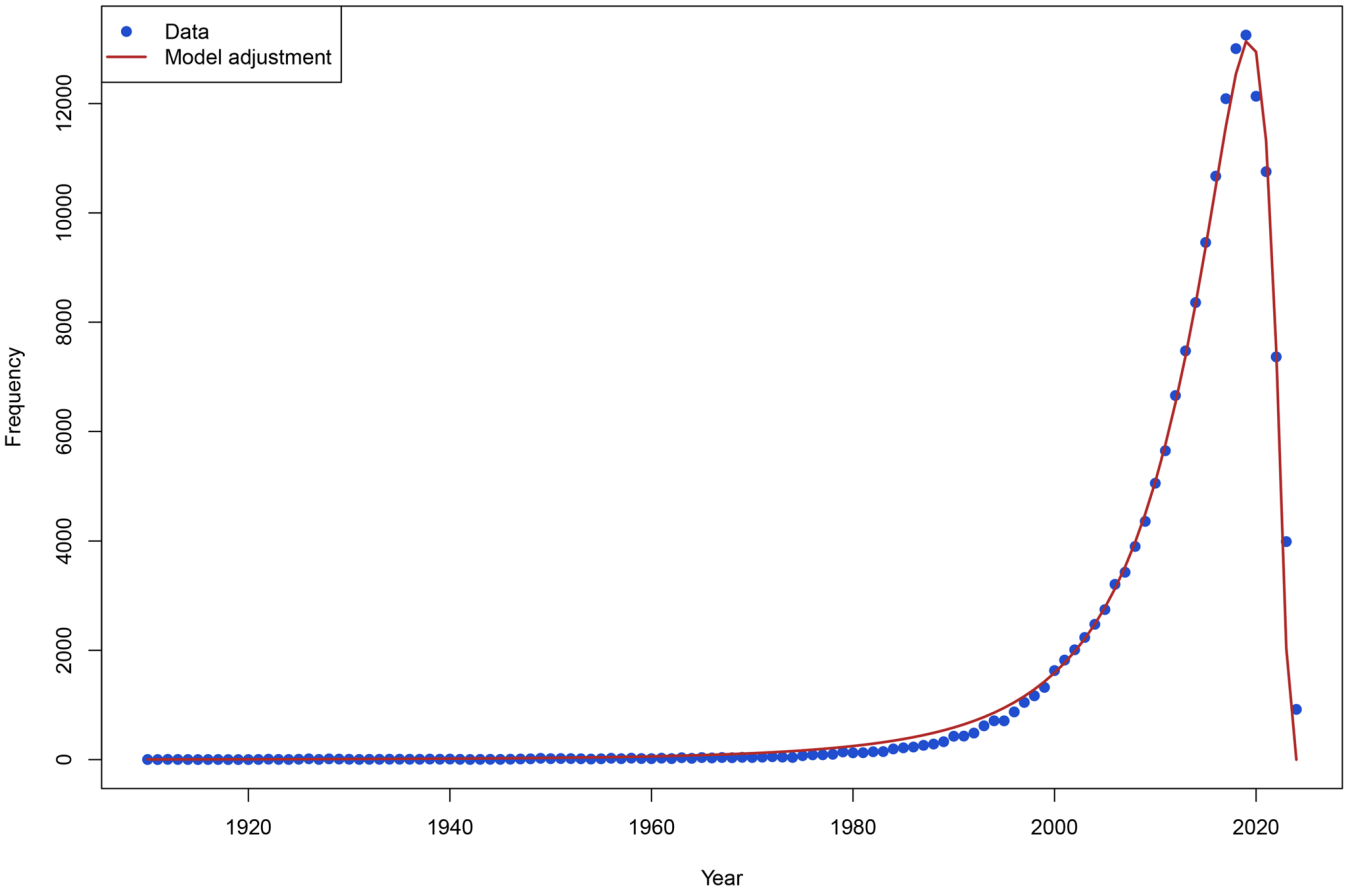
Cited only publications by year. Source: Elaborated following [61].

**Figure 3:**
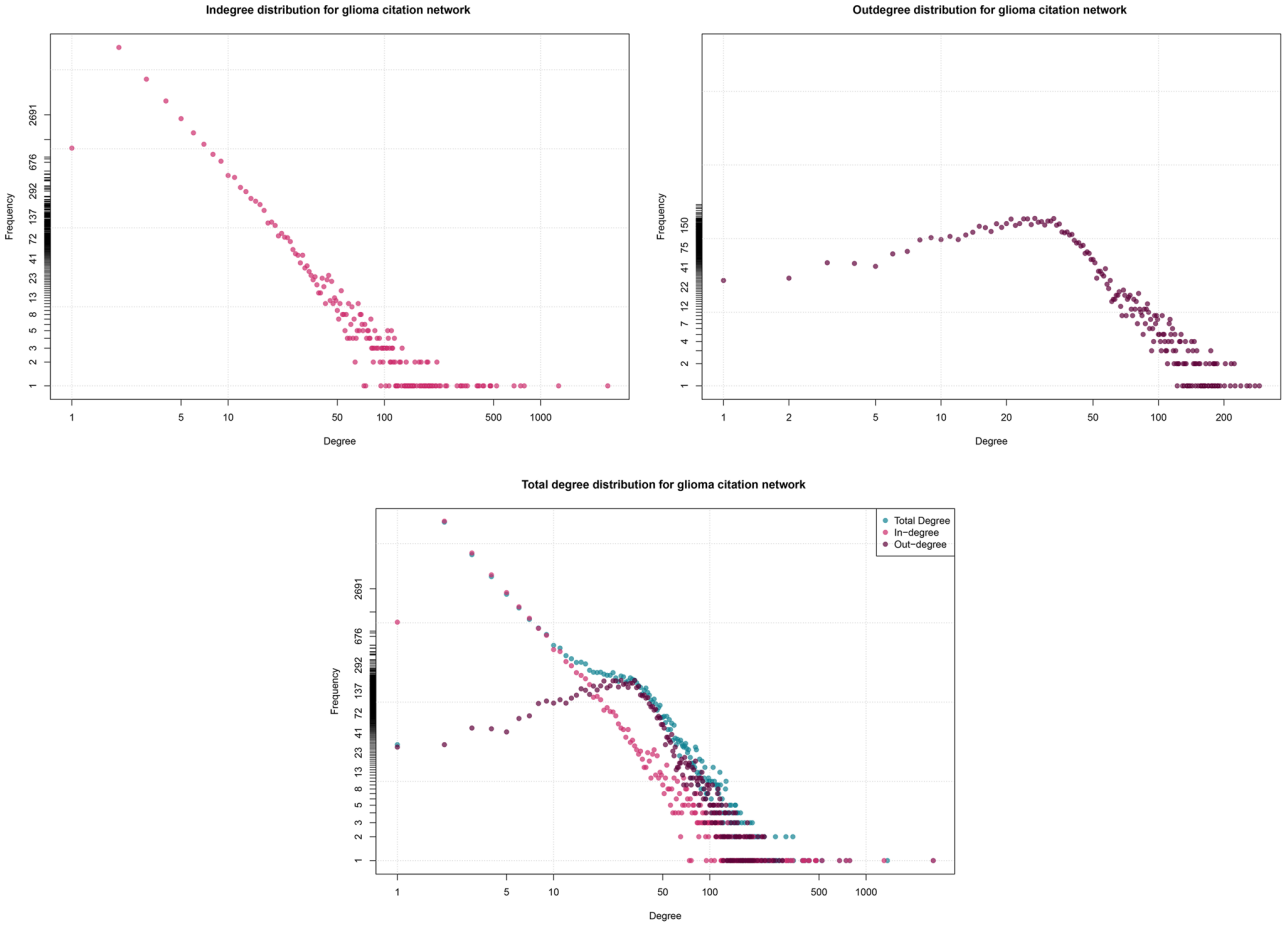
In, out, and total degree distribution (log-log) for G_1_. Source: Elaborated following [61].

Reporting the distribution of cited publications by year (Figure 2) and the network’s degree distribution (Figure 3), provides a basis for the most relevant vertices [59]. It is worth mentioning that Figure 2 does not seek to illustrate only the annual scientific output on gliomas but to reflect the temporal profile of the works that make up the direct citation network. This distinction is necessary because the objective is to understand how recent or how old the literature is, that forms the intellectual core of current research that underpins a particular field, i.e., the relative antiquity of the works that are used as a knowledge base [13, 62]. The time window choice is based on the suggestions of [68] and [69], who emphasized the need to analyze the recent state of the art without losing the historical link through direct citation networks. Thus, the network was not only shaped by the selected time window but also included all the references cited by these articles, thus covering a much broader time spectrum. Figure 2 shows a log-normal fit. This choice was based on the contributions of [70] and [55], who agree that when studying bibliographic networks, scientific works’ citations accumulated over time tend to follow log-normal distributions. In this regard, [62] indicated that this is a typical emergent property in scientific citation networks, where a small fraction of seminal works continues accumulating citations for decades while most recent works have not yet reached citational maturity. This asymmetric distribution is what characterizes log-normal distributions [71]. This phenomenon, combined with documentary obsolescence [63], generates a log-normal distribution of the ages of cited references.

#### Main path analysis

Applying MPA allows us to identify the main evolutionary path of a given field by revaluing the sequence of articles that have had the most significant cumulative impact on the configuration of the scientific network [58]. This is considered relevant for areas such as the study of gliomas, where knowledge evolves rapidly. The following notations describe the process:

- Considering *G* = (*V*, *E*) as a directed graph where:
  - (*V*) set of vertices (scientific articles).
  - (*E*) set of directed edges (citations), where a directed edge from (*v_i_*) to (*v_j_*) is denoted as (*v_i_*,*v_j_*).
- A path in *G* is the sequence of edges:
  $$p=\{(v_{1},v_{2}),(v_{2},v_{3}),\ldots ,(v_{n-1},v_{n})\}\;(1)$$
- If we consider *S* ⊆ *V* as the set of source vertices without predecessors and *T* ⊆ *V* as the set of sink vertices without successors. In this case, each relation *e* = (*v_i_*, *v_j_*) ∈ *E* is assigned a transit weight *w*(*e*) that considers a displacement criterion and its frequency of occurrence in the trajectories. In this sense, the Search Path Count (*SPC*) can be defined as:
  $${\text{w}}_{\text{SPC}}({\text{v}}_{\text{i}},{\text{v}}_{\text{j}})=\sum_{\text{s}\in \text{S}}\sum_{\text{t}\in \text{T}}{\text{δ}}_{\text{st}}({\text{v}}_{\text{i}},{\text{v}}_{\text{j}})\;(2)$$
  Where δ_st_ (v_i_,v_j_) suggests the number of directed search paths from *s* to *t* in which the relation (*v_i_*,*v_j_*) is present at least once.
- Once the weights *w_SPC_* are assigned to all edges, the path p^*^ maximizing the sum of the sum of the weights of the edges that compose it is sought. Subsequently, the path p^*^ ∈ *P* containing the consecutive edges {e_1_, e_2_, …, e_k_} can be obtained by:
  $$p^{*}=\arg\underset{\text{p}\in \text{P}}{\max}\sum_{\text{e}\in \text{p}}{\text{w}}_{\text{SPC}}(\text{e})\;(3)$$
  We utilized Pajek [61] to calculate the SPC weights, which represent the set containing all directed, connected paths. The specific routine used was: Network > Acyclic Network > Create weighted Network + Vector > Transversal Weights > Search Path Count.

#### Key route analysis

Employing KRA in directed networks complements MPA by identifying multiple critical paths, i.e., it not only includes the main path but also identifies parallel paths of high topological relevance that can connect key themes or methodologies [4], which is especially useful in interdisciplinary research where parallel or complementary lines of research may coexist. In the context of the citation network, we considered the following:

- Having transformed the network into an acyclic network and weighted it using path weights *w*(*e*) assigned to each edge *e* = (*v_i_*, *v_j_*) ∈ *E* in E from SPC, the weights quantify the importance of each citation in terms of the flow of trajectories that pass through it, where *w*: *E* → *R*^+^ is the function that indicates that a path weight is assigned to each edge.
- Based on the weighted network, the KRA first identifies a set of key edges, i.e., those with the highest weights. The edges are then sorted by weight in non-ascending order:
  $$w(e_{1})\geq w(e_{2})\geq \cdots \geq w(e_{\left|E\right|})\;(4)$$
  The set of key edges are written as:
  $$L=\{e_{1},e_{2},\ldots ,e_{L}\}\subseteq E,\;(5)$$
  where *L* represents the number of key routes indicated, which in our case was *L* = 10, thus, for each edge considered key e_l=(u_l, v_l)∈L, we aimed to form or construct the associated route by locally extending the trajectory forward and backward, considering the edge with the most significant weight. In general, we obtained: (a) an ascending path $p\_l^{-}$ that connects source nodes in *S* and (b) a descending path $p\_l^{+}$ that seeks to connect $v\_l^{-}$ with sink nodes in *T* by iteratively selecting the edge with the highest *w*(*e*). Thus, by concatenating these segments, a complete directed path (p_l) is produced for each key edge, which provides and traverses the sequence of relevant articles from the perspective of citation flow. Therefore, the set of key edges could be defined as:
  $$P_{key}=\{p\_l|e\_l\in L\}\subseteq P\;(6)$$
  where *P* is the set of all directed paths in the network, in contrast to the global MPA (which produces a single main path), the KRA produces a set of paths that seeks to ensure the inclusion of the most significant links in terms of path weights to reveal patterns of divergence and convergence in the evolution of a given network. Appendix A shows the steps used in Pajek for this process.

#### K-core analysis

The *k – core* decomposition approach was applied to identify densely interconnected substructures [72]. These structures usually correspond to consolidated scientific communities with theoretical and methodological frameworks or research agendas [72]. Considering that the *k* – *core* of a graph *G* denoted as *G_k_* = (*V_k_*, *E_k_*), is the induced subgraph of *G* such that:


$$V_{k}=\{v\in V|\deg_{G_{k}}(v)\geq k\},\;(7)$$

$$E_{k}=\{(u,v)\in E|u\in V_{k},v\in V_{k}\}\;(8)$$

Where deg_*G_k_*_ (*v*) is the degree of the vertex (*v*) in the subgraph *G_k_*, i.e., the number of edges in *G_k_* connected to the vertex (*v*).

Intuitively, to find the k-core of a graph, the process can be understood as follows:

- Remove all vertices in the graph *G* with a degree less than *k*.
- After each removal, recalculate the degrees of the remaining vertices.
- Iterate until no vertices are left with a degree less than *k*.

#### Standardization of citation indicators by year and field

In addition to the citation count, normalized citation indicators at the article level were considered to estimate potential biases that could affect the construction of the main path. If *TC_i_* is considered as the total number of citations for an article *i* that has been published in year *y*(*i*) in category *f*(*i*). For each year *y*, the mean and standard deviation of citations were calculated:


$$\mu_{y}=mean\{TC_{i}:y(i)=y\},\sigma_{y}=standard\;deviation\{TC_{i}:y(i)=y\}\;(9)$$

From this, two standardized annual indicators are defined:


$${\text{rel\_TC\_year}}_{\text{i}}=\frac{{\text{TC}}_{\text{i}}}{{\text{μ}}_{\text{y(i)}}},{\text{z\_TC\_year}}_{i}=\frac{{\text{TC}}_{\text{i}}-{\text{μ}}_{\text{y(i)}}}{{\text{σ}}_{\text{y(i)}}}\;(10)$$

Similarly, for each field–year combination (*f, y*), the following was obtained:


$$\begin{array}{l} {\text{μ}}_{fy}=\text{mean}\{TC_{i}:f(i)=f,y(i)=y\},\\ {\text{σ}}_{fy}=\text{standard deviation}\{TC_{i}:f(i)=f,y(i)=y\} \end{array}\;(11)$$

Furthermore, the standardized indicators were defined by field and year as:


$${\text{rel\_TC\_fy}}_{\text{i}}=\frac{TC_{i}}{\mu_{f(i)y(i)}},{\text{z\_TC\_fy}}_{i}=\frac{TC_{i}-{\text{μ}}_{f(i)y(i)}}{{\text{σ}}_{f(i)y(i)}}\;(12)$$

Following the methodological framework described above, we present the results of the bibliometric analysis, emphasizing key patterns and development trajectories in research on glioma classification. The command lines in Pajek used to obtain the results are provided in Appendix A.

## RESULTS

Figure 2 illustrates the temporal distribution of cited scientific publications. This analysis provides an understanding of the temporal patterns of scientific production and its impact on the network of specific topics. The early phase (early 20^th^ century to the 1960s) suggests a moderate growth in citation frequency, which may be typical in systems where the diffusion of knowledge follows a sigmoidal pattern in the early stages. A low growth rate characterizes this period due to the low connectivity between authors corresponding to that period. From 1960 onwards, the citation rate grew, likely due to factors such as an increase in the number of researchers in the field and a rise in article production with the emergence of new journals.

Figure 2 also identifies a decrease in citation frequency after 2020. While this trend might initially suggest a saturation point, we acknowledge that this pattern must be interpreted with caution, as this decline could be attributed to recency effects, i.e., that more recent publications may not have sufficient time to accumulate citations, rather than an actual stagnation of research output. To mitigate this issue and avoid misleading trends due to incomplete citation data, we restricted our analysis to articles published until 2022. This methodological adjustment follows best practices in longitudinal bibliometric studies [67], where the inclusion of very recent data may distort temporal trends due to the lag in citation accumulation.

Additionally, we modeled the temporal distribution of citations using a log-normal function. The choice of this distribution is based on empirical evidence from prior bibliometric research [57], which has shown that the spread of scientific citations over time often follows a right-skewed distribution, where citation accumulation increases rapidly before reaching a peak and subsequently declines. The log-normal model effectively captures this asymmetric growth-decay pattern, aligning with established citation dynamics observed in large-scale scientific networks. This distribution has been widely employed in similar analyses to quantify the concentration of citations over time and to provide insights into how knowledge accumulates and diffuses within scientific fields [68, 73].

Formally, the probability density function of a log-normal distribution for a random variable *X* is given by:


$$f_{X}(x;\mu ,\sigma )=\frac{1}{x\sigma \sqrt{2\pi }}\exp\left(-\frac{{\left(\ln x-\mu \right)}^{2}}{2\sigma^{2}}\right),\;\;x>0\;(13)$$

Where *μ* and *σ* are the mean and standard deviation of the variable in the logarithmic space. Applying the model to the context of this paper, to model citation frequency as a function of year (*y*) and assuming that *X* = 2022 − *y* fits a log-normal distribution, *freq*(*y*) could be expressed by:


$$\text{freq}(y)=c\times \frac{1}{(2022-y)\cdot b\cdot \sqrt{2\pi }}\exp\left(-\frac{{\left(\ln(2022-y)-a\right)}^{2}}{2b^{2}}\right)\;(14)$$

where:

- *c* = 164631.296 is the scaling coefficient; it acts as a multiplicative factor that adjusts the width of the distribution to align it with the observed empirical frequencies.
- *a* = 2.034 location parameter, the mean of the logarithm of the variable (year shifted) in the distribution. In this context, it reflects the average year (shifted from 2022) when the citation frequency reaches its maximum. As the model is centered on 2022, the maximum average year is 2019–2020.
- *b* = 1.033 scaling parameter (standard deviation in log space), an indicator of the dispersion of citations around the peak year.
- 2022 − *y* and temporal transformation that centers the distribution on the year 2022.

Simplifying (8) the model could be expressed as:


freq(y)=c×LogNorm(2022−y; a, b) (15)

We used Pajek [62] to produce some graphs. The first directed graph, *G*_1_ = (*V*_1_, *E*_1_), represents the citation network between scientific papers. In this graph, vertices *v* ∈ *V*_1_ represent scientific papers. There is a directed relation (*v*, *u*) ∈ *E*_1_ if paper *u* has cited paper *v*. This allows for analyzing the influence and significance of scientific papers by illustrating their citations of each other, thereby identifying the most influential papers and the connections between different research.

*G*_1_ contains 46,204 vertices and 231,432 arcs, with an average degree of 10.0178. This suggests that, on average, each paper in the network cites or is cited by approximately 10.01 other papers. The citation graph remains sparse, with a density of 0.00010841.

In Figure 3, the degree distributions adhere to a power-law distribution, i.e., the likelihood of a node having a degree *k* is defined by a function of the form *P*(*k*) ~ *k^−γ^*, with *γ* as a positive exponent, with most nodes having low degrees and a few nodes exhibiting high degrees, reflecting the diversity of the literature in glioma classification. The high in-degree, peaking at 2691, indicates the presence of influential works that significantly impact the field, while the out-degree of 291 suggests a broad bibliographic scope, though over a thousand relevant articles are not included in the review process.

Table 3 highlights the 50 articles with the most citations, many from recent years (2019–2022), with Krichevsky’s 2019 paper [74] leading in citations (approximately 13%), focusing on oligonucleotide-based therapies for brain tumors. Other key articles include those by [11, 75, 76], and [77], which were cited 2278, 263, 255, and 239 times, respectively (accounting for 44% of citations), together with Krichevsky’s work, represent interdisciplinary studies integrating knowledge on glioma therapies, molecular pathways, and immunotherapeutic strategies.

**Table 3:** Articles that most cite other papers on *G*_1_ (outdegree based)

Rank	Frequency	Id	Rank	Frequency	Id
1	291	KRICHEVS_A(2019)16:319	26	179	SABU A(2022):2c03538
2	278	YANG K(2022)21:s12943-022-01513-z	27	179	CHEN S(2021)26:417
3	263	WELLER M(2024)10:s41572-024-00516-y	28	178	SCHEIE D(2019)127:265
4	255	IUS T(2023)162:267	29	176	BALANA C(2022)13:865171
5	239	SLEDZINS P(2021)22:ijms221910373	30	175	MOREAU A(2019)9:01134
6	225	BUCHLAK Q(2021)89:177	31	174	PICCA A(2023)23:1217
7	223	ONCIUL R(2024)46:2402	32	174	MIRCHIA K(2020)12:cancers12071817
8	223	MELHEM J(2022)19:1705	33	174	SHAHZAD U(2021)13:cancers13071555
9	216	MUZYKA L(2023)24:ijms241310456	34	172	GUZMAN G(2023)196:76
10	216	VEGA J(2018)25:143	35	172	ROOSEN M(2022)143:427
11	214	SHIKALOV A(2024)16:pharmaceutics16010100	36	170	ALGHAMRI M(2021)12:680021
12	203	D’AMATI A(2024)17:1268038	37	169	LI C(2022)49:E1024
13	203	SHEN Y(2024)13:s40164-024-00512-8	38	165	CIPRI S(2023)13:1204829
14	201	TILAK M(2021)22:ijms22041831	39	165	DEOCESAN C(2020)21:ijms21072611
15	192	PITARCH C(2024)16:cancers16020300	40	164	VANDENBE M(2023)402:1564
16	188	SLEDZINS P(2024)112:63	41	163	LOCARNO C(2020)225:002
17	187	WU P(2023)1405:31	42	162	CUI J(2022)12:1100
18	186	MCNAMARA C(2022)64:1919	43	161	SILANTYE A(2019)8:cells8080863
19	186	BRAT D(2022)146:547	44	160	JOHNSON A(2022)12:995498
20	183	AMIN J(2022)8:3161	45	158	FARES J(2024)6:fcae108
21	182	SHARMA H(2020)151:1	46	157	BAUSART M(2022)41:s13046-022-02251-2
22	182	FERRER V(2018)66:1542	47	156	KIM Y(2021)9:s40478-021-01151-4
23	181	STEPANEN A(2024)15:1326753	48	155	GARGINI R(2020)12:cancers12061622
24	180	SANSONE G(2022)10:517	49	155	GONCALVE F(2021)12:733323
25	180	LU Q(2019)8:342	50	153	AGGARWAL P(2022)13:1038096

Source: Self-elaboration using Pajek [62].

Table 4 highlights the most cited papers in *G*_1_, including [49] paper with 2961 citations, which updates CNS tumor classification and emphasizes the integration of molecular and histological features in glioma classification and prognosis, and [50] paper, which is also highly cited (1304 citations) for providing critical information to neuropathology and oncology specialists, incorporating new tumor types and subtypes, redefining nomenclature, and implementing an integrated reporting system.

**Table 4:** Most cited articles on *G*_1_ (indegree based)

Rank	Frequency	Id	Rank	Frequency	Id
1	2691	LOUIS D(2016)131:803	26	217	OSTROM Q(2019)21:V1
2	1304	LOUIS D(2021)23:1231	27	215	SCHWARTZ J(2012)482:226
3	786	STUPP R(2005)352:987	28	212	WELLER M(2017)18:E315
4	745	BRAT D(2015)372:2481	29	205	CAIRNCRO G(2013)31:337
5	676	YAN H(2009)360:765	30	202	WESSELIN P(2018)44:139
6	523	ECKEL-PA J(2015)372:2499	31	198	BUCKNER J(2016)374:1344
7	481	VERHAAK R(2010)17:98	32	195	HE K(2016):770
8	477	LOUIS D(2007)114:547	33	193	PHILLIPS H(2006)9:157
9	474	CAPPER D(2018)555:469	34	192	NOUSHMEH H(2010)17:510
10	433	CECCAREL M(2016)164:550	35	191	REUSS D(2015)129:133
11	427	MENZE B(2015)34:1993	36	190	OSTROM Q(2018)20:1
12	405	HEGI M(2005)352:997	37	190	HARTMANN C(2010)120:707
13	397	BRENNAN C(2013)155:462	38	187	BRAT D(2020)139:603
14	392	OSTROM Q(2015)16:1	39	187	SUZUKI H(2015)47:458
15	340	PARSONS D(2008)321:1807	40	185	WEN P(2008)359:492
16	330	LOUIS D(2007)114:97	41	184	GILLIES R(2016)278:563
17	318	STUPP R(2009)10:459	42	180	RONNEBER O(2015)9351:234
18	310	BRAT D(2018)136:805	43	179	WANG Q(2017)32:42
19	307	BAKAS S(2017)4:117	44	174	LOUIS D(2018)135:639
20	292	CHIN L(2008)455:1061	45	172	SHIRAHAT M(2018)136:153
21	251	WEN P(2010)28:1963	46	172	SUBRAMAN A(2005)102:15545
22	246	STURM D(2012)22:425	47	171	TURCAN S(2012)483:479
23	229	VANDEN B(2013)31:344	48	168	PATEL A(2014)344:1396
24	224	WELLER M(2021)18:170	49	167	VANGRIET J(2017)77:E104
25	217	OSTROM Q(2014)16:896	50	167	MACKAY A(2017)32:520

Source: Self-elaboration using pajek [62].

Other relevant articles include STUPP_R(2005) 352:987 [78] and BRAT_D(2015)372:2481 [79] with 786 and 745 citations, respectively; the former is pioneering in establishing the combined regimen of radiotherapy with temozolomide as an effective treatment for glioblastoma, while the latter revolutionized glioma classification by incorporating genomic data and identifying molecular subtypes that more accurately predict clinical behavior than traditional histology. Additionally, YAN_H(2009)360:765 [80] (676 citations) confirmed the survival benefit of adding temozolomide to radiotherapy in glioblastoma and ECKELPA_J(2015)372:2499 [81] (523 citations) advanced the molecular classification of gliomas through the identification of mutations in TERT, IDH and 1p and 19q codeletion.

### Main path analysis

MPA made it possible to reconstruct the sequence of works that have accumulated the greatest flow of citations within the network, and with this, it is possible to say that the intellectual backbone or the central evolutionary trajectory that has shaped the field of research on glioma classification was identified. In this respect, this section not only describes the biomedical content of each work but also seeks to explain the way in which these works have acted as key nodes linking concepts, methodologies, and approaches.

**Figure 4:**
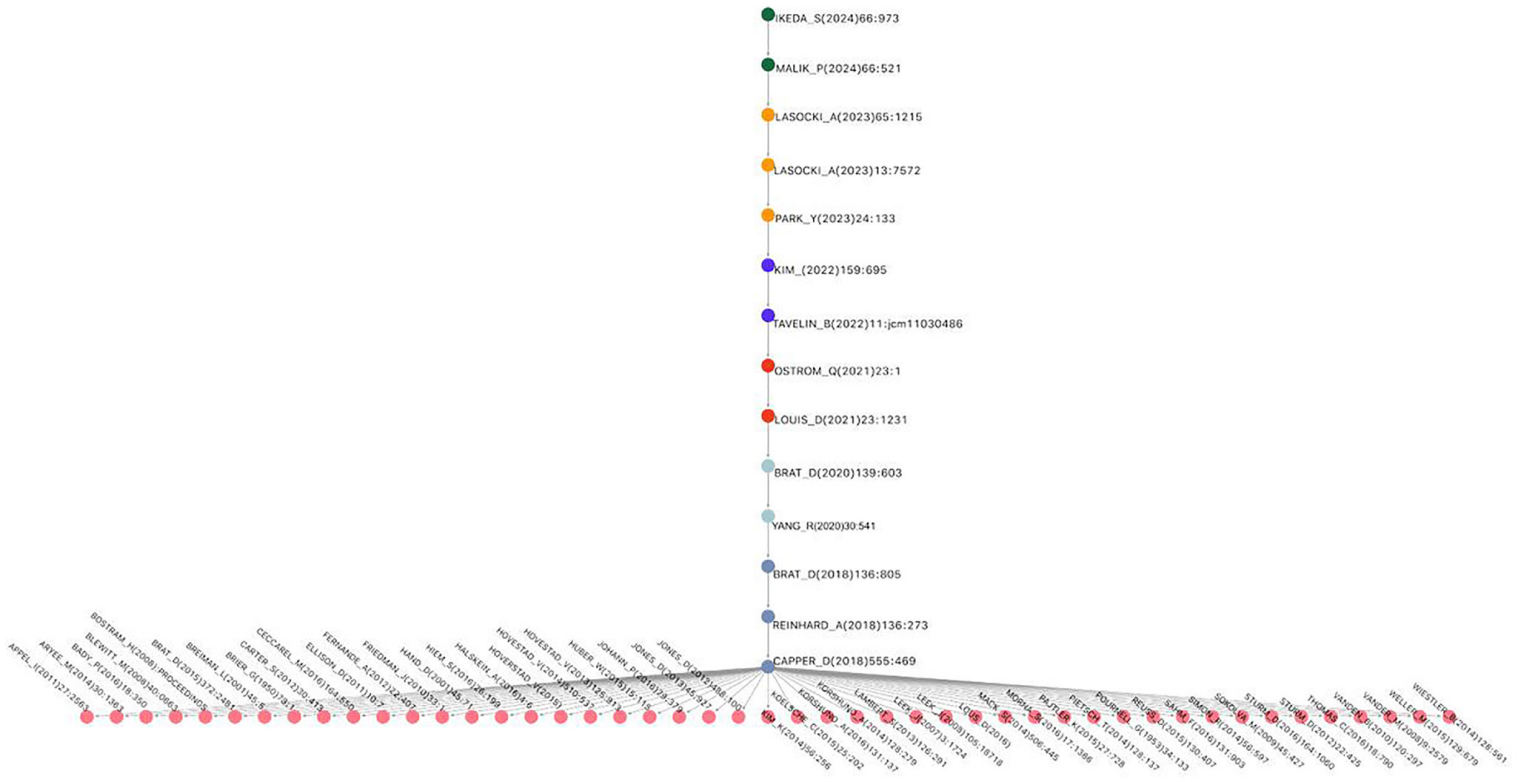
Main Path for glioma research network. Source: Elaborated following [62].

Figure 4 shows the main path of research on glioma classification. Each node denotes a seminal work, and the directional edges trace the chronological and thematic progression. This visualization seeks to highlight how a subset of works underpins the intellectual structure of the field, and to improve the clarity and readability of the results, Table 5 summarizes the key findings of the MPA. This table not only presents the papers that make up the main route but also emphasizes the specific bibliometric role of each one within the citation network. Instead of offering only a biomedical description of the articles, this synthesis highlights how each work acts as a key node that links concepts, methodologies, and approaches, outlining the theoretical and technical evolution in the classification of gliomas. Thus, the table shows in a structured way how the knowledge network has evolved, from the introduction of molecular classification to the integration of clinical, epidemiological, and imaging data. This format allows us to more clearly appreciate how the citation flow reflects the process of consolidation and expansion of a scientific paradigm, thereby fulfilling the central purpose of bibliometric analysis.

**Table 5:** Main path analysis: Frameworks of research on glioma classification

Source	Methodological contribution	Role in main path	Clinical correlates
Acta Neuropathologica [51]	DNA methylation and Random Forest classification.	Initiates molecular paradigm shift.	Redefines glioma taxonomy.
Acta Neuropathologica [82]	t-SNE and clustering for molecular classification.	Refines classification for subgroups.	Adapts diagnostics for rare gliomas.
Acta Neuropathologica [83]	Molecular biomarkers in IDH-wildtype glioma.	Bridges molecular biology and clinical practice.	Enables molecular-based prognosis.
Brain Pathology [84]	PDGFRA, CDKN2A, CDK4 biomarkers for IDH-mutant gliomas.	Enhances risk stratification.	Improves prognosis tools.
Acta Neuropathologica [85]	Updates WHO IDH-mutant astrocytoma guidelines.	Institutionalizes molecular classification.	Standardizes clinical decisions.
Neuro-Oncology [50]	Integrates molecular and histological WHO criteria.	Establishes a global classification.	Sets glioma diagnostic reference.
Neuro-Oncology [86]	Links molecular classification with epidemiology.	Connects taxonomy with public health.	Provides incidence and survival data.
Journal of Clinical Medicine [87]	Sex disparities in glioblastoma outcomes.	Expands classification with demographics.	Highlights sex as a prognostic factor.
Journal of Neuro-oncolgy [88]	Sex-specific glioma survival with molecular markers.	Combines molecular and demographic data.	Improves personalized treatment.
Korean Journal of Radiology [89]	MRI biomarkers for CDKN2A/B deletion.	Links molecular and imaging markers.	Advances non-invasive diagnosis.
Journal of Medical Imaging and Radiation Oncology [23]	MRI and molecular data integration.	Strengthens multimodal diagnostics.	Promotes imaging-genetics synergy.
Neuroradiology [90]	T2-FLAIR patterns and glioma subtypes.	Reinforces imaging molecular integration.	Enhances pre-surgical assessment.
Neuroradiology [91]	Clinical/imaging predictors for IDH2 mutation.	Bridges molecular, imaging, and clinical data.	Improves early diagnosis.

In addition, we verified that the identified main path was not solely the result of the selected weighting method as we evaluated its robustness on the citation network by recalculating the edge weights using Search Path Count (SPC), Search Path Link Count (SPLC), and Search Path Node Pair (SPNP) in Pajek (Appendix A). Subsequently, we consider *V*^(*w*)^ and *E*^(*w*)^ as the set of vertices and edges, respectively, of the main path subgraph obtained under any of the schemes *w* ∈ *SPC*, *SPLC*, *SPNP*. Therefore, to quantify the degree of coincidence between paths, the Jaccard index was used at both the vertex and edge levels: *J_v_*(*w*_1_, *w*_2_) = |*V*^(*w*_1_)^ ∩ *V*^(*w*_2_)^|/|*V*^(*w*_1_)^ ∪ *V*^(*w*_2_)^| and *J_E_*(*w*_1_, *w*_2_) = |*E*^(*w*_1_)^ ∩ *E*^(*w*_2_)^|/|*E*^(*w*_1_)^ ∪ *E*^(*w*_2_)^|, where values close to 1 would indicate almost complete overlap between the compared structures. The results suggest that the three networks contain the same set of 61 articles and 60 edges, that is, *V*^(*SPC*)^ = *V*^(*SPLC*)^ = *V*^(*SPNP*)^ and *E*^(*SPC*)^ = *E*^(*SPLC*)^ = *E*^(*SPNP*)^. Therefore, the Jaccard index is equal to 1 for both vertices and artists in the comparisons SPC vs. SPLC, SPC vs. SPNP, and SPLC vs. SPNP, implying that the main path is invariant with respect to the choice of path weighting scheme. Additionally, we seek to rule out or evaluate whether the route obtained using Pajek could be biased toward specific works with high citation impact, even after normalizing citations by year and by field/year. In this sense, we associated each article with four normalized citation indicators (*rel_TC_year_*, *z_TG_year_*, *rel_TC_fy_* and *z_TC_fy_*) to compare the average values between the works that make up the main path and the rest of the network (Table 6).

**Table 6:** Normalized citation indicators for papers within and outside the main path

Group	*n*	mean_rel_TC_year	Mean_z_TC_year	Mean_rel_TC_fy	Mean_z_TC_fy
Off the main path	6828	0.9997	−0.0002	1.0002	−0.0003
Within the main path	53	1.0268	0.0313	0.9681	0.0429

The identified main path contains 61 vertices. However, the normalized citation analysis was restricted to the 53 articles for which complete metadata were available in the Web of Science Core Collection, including publication year, subject category, and citation counts within the 2018–2024 observation window. The remaining eight vertices correspond to cited references that do not have source records indexed in Web of Science; therefore, they were excluded from the computation of normalized citation indicators.

The results in Table 6 suggest that the items outside the main path showed mean values and z-scores close to zero (rel_TC_year ≈ 1.00, z_TC_year ≈ 0.00; rel_TC_fy ≈ 1.00, z_TC_fy ≈ 0.00), while the items that make up the main path exhibited slight deviations (rel_TC_year ≈ 1.03, z_TC_year ≈ 0.03; rel_TC_fy ≈ 0.97, z_TC_fy ≈ 0.04 in absolute value). When considering the results together, it is possible to say that the differences are small in terms of standard deviation, suggesting that the main path was not formed exclusively by extraordinary works, as indicated by their normalized citations, but rather by articles whose impact is comparable to the field average. In other words, their identification responds primarily to the structure of the citation network rather than to biases in relative impact metrics.

### Key route analysis

The application of KRA to the citation network of glioma classification revealed a more complex knowledge flow than the single evolutionary track identified through MPA. While MPA highlighted the central intellectual backbone, KRA exposed multiple complementary trajectories that reflect parallel contributions from different research approaches. These complementary routes capture methodological innovations, clinical translation efforts, and conceptual refinements that collectively shaped the evolution of glioma classification research.

**Figure 5:**
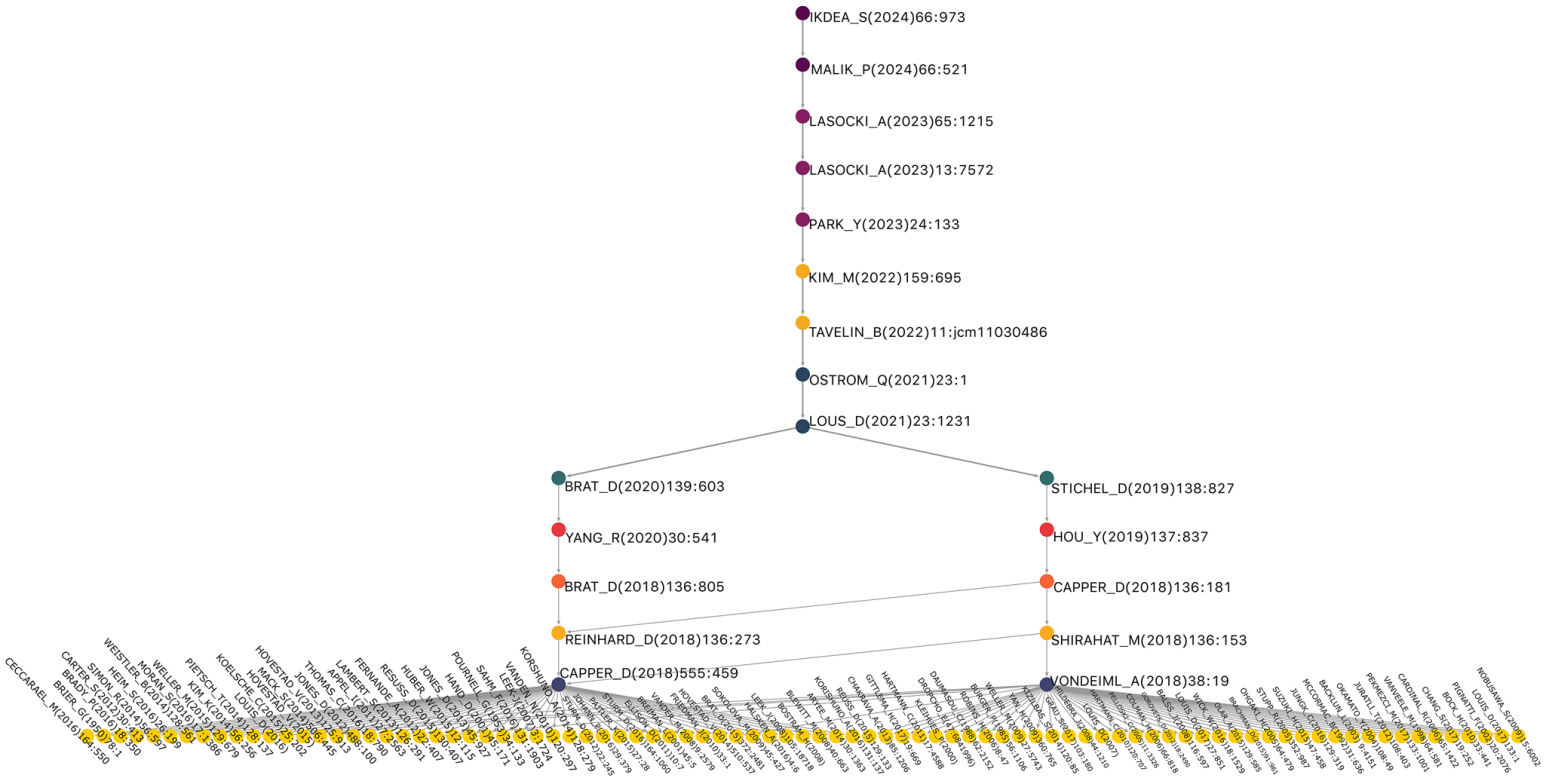
Key route for the glioma research network. Source: Elaborated following [62].

Figure 5 illustrates KRA, revealing complementary trajectories running parallel to the main pathway. These pathways highlight methodological innovations, such as the advanced integration of genetics and imaging, and show how they intersect with the dominant molecular paradigm. The figure also demonstrates advances in glioma classification arise from multiple converging streams. This key route consists of 106 nodes, of which 14 replicate the core main path already shown in Figure 4 (starting with CAPPER_D(2018)555: 469→BRAT_D(2020)139:603 following right to LOUIS_D(2021)23:1231→IKEDA_S(2024)66:973. This overlap indicates that the main path acts as the central spine, while the key route expands outward, incorporating additional influential works that interact with and enrich the primary intellectual trajectory.

In addition to the main sequence, the KRA identifies a second key source node, represented by the work of VONDEIML_A(2018)38:19, which links early morphology-based classification approaches with more recent molecular methods, highlighting a transitional phase in the evolution of glioma diagnostics. This integration of historical and contemporary methodologies is central to understanding how glioma classification evolved into its current form.

Table 7 summarizes the key works identified in the KRA. This table is structured to emphasize how each work contributes not only to scientific knowledge but also to clinical translation, reinforcing the bibliometric narrative that glioma classification has progressively incorporated molecular and imaging data into routine clinical practice.

**Table 7:** Key route analysis: Complementary research on glioma classification

Source	Methodological contribution	Role in main path	Clinical correlates
Acta Neuropathologica [51]	DNA methylation and Random Forest classification.	Initiates molecular paradigm shift.	Redefines glioma taxonomy.
Acta Neuropathologica [82]	t-SNE and clustering for molecular classification.	Refines classification for subgroups.	Adapts diagnostics for rare gliomas.
Acta Neuropathologica [83]	Molecular biomarkers in IDH-wildtype glioma.	Bridges molecular biology and clinical practice.	Enables molecular-based prognosis.
Seminars in Neurology [92]	Evolution from histological to molecular classification.	Links past and modern paradigms.	Improves diagnostic accuracy.
Acta Neuropathologica [93]	CDKN2A/B deletion and CNV load for grading.	Adds genomic criteria to classification.	Enhances risk stratification.
Acta Neuropathologica [94]	Molecular diagnostics in clinical routine.	Validates real-world applicability.	Improves diagnostic reliability.
Acta Neuropathologica [95]	Addresses misclassification due to morphology.	Highlights value of molecular profiling.	Reduces diagnostic errors.
Acta Neuropathologica [52]	RNA sequencing in FFPE samples.	Expands molecular pathology toolkit.	Provides new therapeutic targets.
Brain Pathology [84]	PDGFRA, CDKN2A, CDK4 biomarkers for IDH-mutant gliomas.	Enhances risk stratification.	Improves prognosis tools.
Acta Neuropathologica [85]	Updates WHO IDH-mutant astrocytoma guidelines.	Institutionalizes molecular classification.	Standardizes clinical decisions.
Neuro-Oncology [50]	Integrates molecular and histological WHO criteria.	Establishes a global classification.	Sets glioma diagnostic reference.
Neuro-Oncology [86]	Links molecular classification with epidemiology.	Connects taxonomy with public health.	Provides incidence and survival data.
Journal of Clinical Medicine [87]	Sex disparities in glioblastoma outcomes.	Expands classification with demographics.	Highlights sex as a prognostic factor.
Journal of Neuro-oncolgy [88]	Sex-specific glioma survival with molecular markers.	Combines molecular and demographic data.	Improves personalized treatment.
Korean Journal of Radiology [89]	MRI biomarkers for CDKN2A/B deletion.	Links molecular and imaging markers.	Advances non-invasive diagnosis.
Journal of Medical Imaging and Radiation Oncology [23]	MRI and molecular data integration.	Strengthens multimodal diagnostics.	Promotes imaging-genetics synergy.
Neuroradiology [90]	T2-FLAIR patterns and glioma subtypes.	Reinforces imaging molecular integration.	Enhances pre-surgical assessment.
Neuroradiology [91]	Clinical/imaging predictors for IDH2 mutation.	Bridges molecular, imaging, and clinical data.	Improves early diagnosis.

It is worth mentioning that the comparative analysis between MPA and KRA shows that both techniques respond to different analytical objectives and generate complementary results. The MPA identifies a unique and linear trajectory that concentrates the most intense flow of knowledge over time, acting as the intellectual backbone of the field and simultaneously highlighting the central sequence of works that were decisive in the historical configuration of glioma classification. In contrast, KRA recognizes that scientific progress rarely follows a single linear path, so this technique reflects how different lines of research connect to build a cohesive body of knowledge. Thus, KRA allows capturing the dynamics of convergence and bifurcation that characterize highly complex fields, such as molecular oncology; hence, the combination of MPA and KRA is relevant for this study as it attempts to reflect more accurately the interdisciplinary and incremental nature of glioma research.

### *K*-core analysis

*K – core* analysis analysis helps identify highly connected substructures which can offer valuable insights into cohesive research communities and relevant articles. In this work, we applied this technique to a citation network of scientific articles on glioma classification. This is considered relevant as it allows us to identify influential hubs or scientific communities regarding density and connectivity. This approach allows us to understand the internal structure of knowledge in the context of the network articles and highlight nodes or articles that play a critical role in disseminating topics or methodological approaches and in the connection between scientific disciplines.

The maximum number of cores for this subgraph is 19 (Table 8). Once the network cores had been identified, extracting the *core* corresponding to the highest value of *k* was displayed graphically, ensuring that the drawing had no more than one hundred nodes to avoid overload.

**Table 8:** K-cores in glioma research network

Cluster	Freq	Freq (%)	CumFreq	CumFreq (%)	Representative
0	112	0.2424	112	0.2424	LIAO_Y(2020)99:0000000000021002
1	38	0.0822	150	40.9207	CARVER E(2019)21:61
2	18757	40.5961	18907	40.9207	BORMANN F(2018)23:3407
3	7432	16.0852	26339	57.0059	BRANCATO_V(2022)14:cancers14112731
4	4042	8.7482	30381	65.7540	GANESHAN B(2013)266:326
5	2607	5.6424	32988	71.3964	GROVE O(2015)10:0118261
6	1791	3.8763	34779	75.2727	BAILEY O(1986)12:261
7	1522	3.2941	36301	78.5668	KERN M(2020)20:1
8	1216	2.6318	37517	81.1986	KOBAYASH K(2021)11:2
9	1080	2.3375	38597	83.5361	SAWLANI V(2020)11:1
10	951	2.0583	39548	85.5943	KOELSCHE C(2021)12:4
11	871	1.8851	40419	87.4794	PROVENZA J(2006)239:632
12	853	1.8462	41272	89.3256	SHUKLA G(2017)6:28
13	824	1.7834	42096	91.1090	DAVNALL F(2012)3:573
14	807	1.7466	42096	92.8556	ARONEN H(1994)191:41
15	867	1.8765	43770	94.7321	LIU J(2018)173:400
16	853	1.8462	44623	96.5782	CINARER G(2020)10:app10186296
17	478	1.0345	45101	97.6128	GAO M(2020)10:01676
18	743	1.6081	45844	99.2208	CHO H(2018)6:5982
19	360	0.7792	46204	100.0000	LOUIS D(2021)23:1231

Source: Elaborated based on [62].

Table 8 presents the results of the citation network in the context of our work. It should be recalled that *k – core* are maximal subgraphs where each node, in this case article, has at least *k* connections to other vertices. We find a range of cores from *k* = 0 to *k* = 19, with 46,204 nodes analyzed. The largest proportion of articles is found at *k – core* = 2 (representing 40.59% of the nodes), suggesting that a substantial portion of the works are weakly connected, i.e., most nodes have at least two connections but fail to achieve high levels of cohesion.

Since we are looking for the most cited articles within the network, we focus on cluster 19, which represents articles with the highest number of citations that are considered key references or reviews in glioma research. It contains 360 articles representing 0.7792% of all nodes in our network. Given that the vertices of this subgraph have at least 19 connections to other nodes in the network, this could indicate that these articles are not only relevant because of their high citation counts but may also be fundamental to the dissemination of crucial knowledge and methodologies in the study of gliomas. Their removal could negatively affect the network’s cohesion and structural integrity.

For the selection of *k*, we considered different subgraph sizes, selecting those with a coreness greater than or equal to *k*. Based on the partition, the maximum coreness obtained was 19. Hence, a *k*-core = 15 or equal to 18, the size of the induced graph is gradually reduced from 3,301 nodes (7.14% of the network) to 1,103 vertices (2.39%). In the same vein, moving from k-core = 18 to 19 resulted in a contraction to 360 vertices, indicating that *k* = 19 yields a non-trivial, structurally relevant core. Additionally, the robustness of the selected core was evaluated considering threshold variations by recalculating k in a range [15, 25] to review its composition, observing that in a feasible range *k* ≤ 19, nodes with coreness 19 are part, by definition, to the lower-order k-core in such a way that the articles that define the paradigmatic core are also present in *G_k_* for *k* = 15–18, and what changes with the decrease in *k* is that works with a better degree of structural incrustation are incorporated. The above can be expressed as *N*_19_ = *v*: *coreness*(*v*) = 19. It can be verified that *N*_19_ ⊆ *G_k_* for all *k* ∈ {15,16,17,18,19} and that, in *k* > 19, the core is empty, so no stricter threshold produces an interpretable substructure.

Based on the above, and from a structural perspective, the choice of threshold k involved capturing the most interconnected core of the field, while relaxing the thresholds expands the graph structure by incorporating more recent works or emerging nodes that have not yet migrated to what can be considered the paradigmatic core. Along these lines, we do not seek to specifically quantify the number of emerging nodes that would enter the k-core under different thresholds or integrate additional altimetric indicators, as this would mean moving away from the methodological design, and we consider these to be future lines of work that could enrich the reconstruction of the paradigmatic core presented.

**Figure 6:**
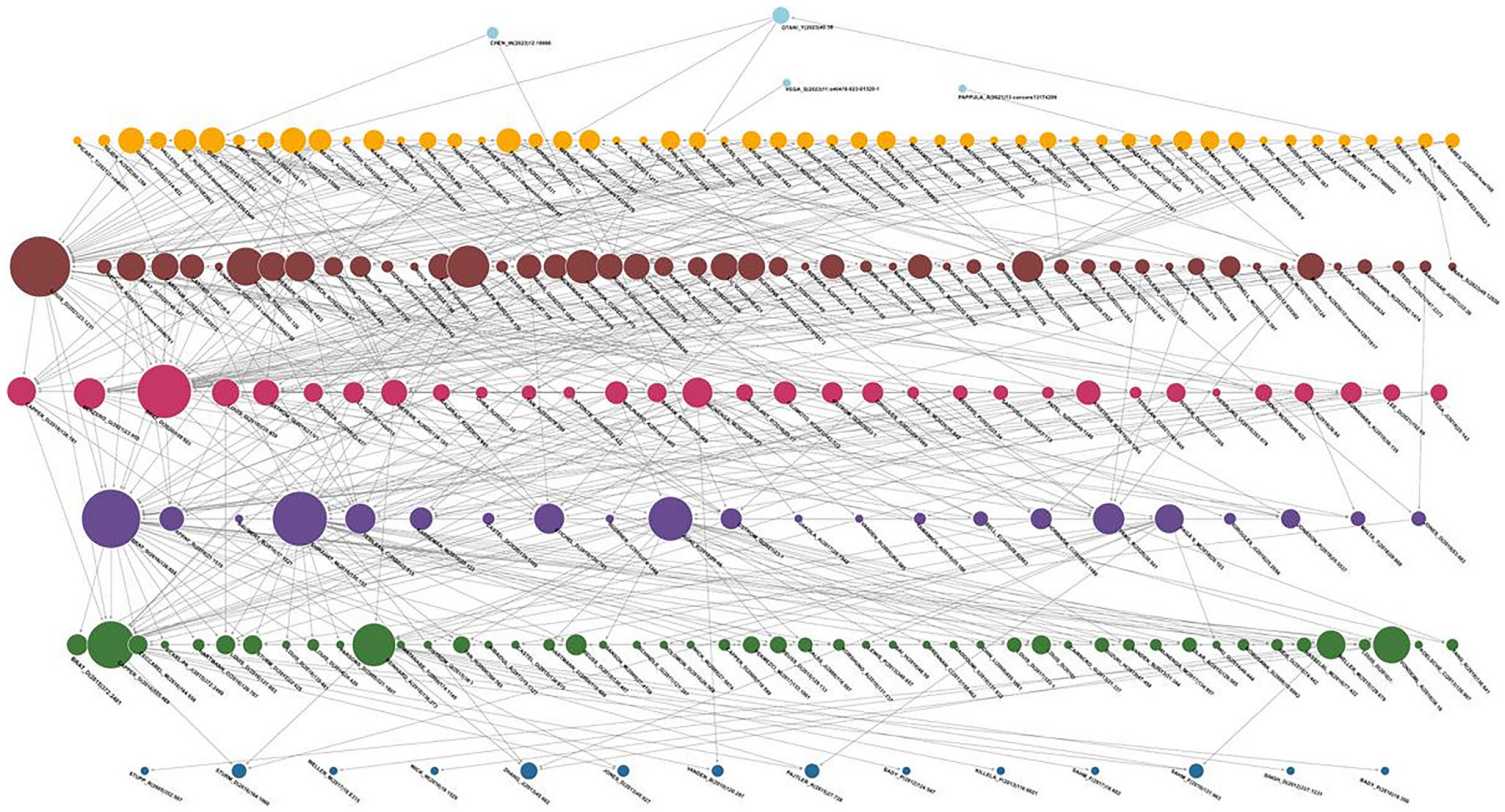
Subgraph based on k-core = 19 for the glioma research network. Source: Elaborated following [62].

Figure 6 shows that the subgraph is highly interconnected. However, all nodes have at least 19 connections to other nodes within the same subgraph, suggesting that each article is highly cited and cites extensively other influential works within the core. This visualization also allows displaying a hierarchy of nodes by different sizes, which was adjusted using the total degree, where larger nodes represent articles with more connections within the *k – core*. The degree of centrality of these articles may suggest systematic reviews, consensus studies, or papers that have introduced new paradigms or methodologies in glioma research. They also serve as connectors of different thematic areas, facilitating collaboration and interdisciplinary development.

Based on the above, the studies by [86] and [50] provide updated frameworks for glioma classification, marking a shift from histological to molecular-based approaches, integrating markers like IDH mutations, 1p and 19q codeletion, and TERT promoter mutations. This evolution in classification is further refined by the work of [94] and [96], which introduced DNA methylation-based methods. The integration of biomarkers, as discussed in [84] and [85], supports the robustness and prognostic value of molecular systems, enabling better patient stratification.

Further advancements in glioma stratification are reflected in studies by [3, 16, 18, 90, 92], and [54], which combine histological, molecular, and morphological data into complex classification algorithms, improving predictive power for clinical outcomes.

## DISCUSSION

In summary, our work sought to deepen and provide a structured view of research focused on the classification of gliomas using network analysis as the primary tool. First, we reviewed the distribution of publications cited by year, which allowed us to identify how knowledge evolved or accumulated through inflection points or periods in which there were significant increases in citations, which, in the context of this work, were found to occur starting in 2002. The first objective was achieved by identifying the distribution of degrees in the direct citation network and found the three most cited articles by [49], [50], and [78] correspond to updates in tumor classification and the impact of radiotherapy application. In addition, the application of MPA and KRA helped identify influential articles and critical connections that allowed us to understand how different areas of knowledge in neuro-oncology have evolved. Unlike the work of [6, 41], and [22], which focused on grouping topics, our results show chronological development and evolution based on the interconnection and influence of methods.

[12, 36, 38], agreed that the impact of studies can only be measured through citation counts or centrality metrics. However, we believe that, if taken as definitive data, these metrics can offer an incomplete perspective of a citation network, leading to inadequate identification of critical articles that act as turning points and influence the adoption of new methodologies or the negotiation and adoption of interdisciplinary approaches. Furthermore, by relying solely on input data, the complexity of knowledge development is overlooked.

We also state that the similarity of our work with the reviewed literature lies in the relevance of introducing molecular biomarkers for the classification of gliomas, as demonstrated by [24, 25, 92] and [94] who have also highlighted the need to go beyond traditional histological techniques, proposing classifications based on DNA methylation and genetic alterations, which are more accurate. The application of MPA reinforces this idea by placing these studies as part of key pathways in the knowledge network, suggesting how this work has facilitated the integration of molecular approaches and driven their incorporation into clinical practice. In contrast, unlike the proposals of [18, 31] and [39], which are limited to identifying prominent authors or collaborative groups, our network approach illustrates how ideas and research efforts are interconnected with emerging subfields, highlighting the relevance of these studies, not only as pioneers of innovative techniques, but also as bridges between methodological and clinical approaches that foster diversification and advancement in the field of glioma classification research.

Our approach to presenting and highlighting connections between articles differs from the findings reported in the bibliometric data we reviewed. For example, studies such as those conducted by [97] and [75] focused their analysis on both the validity of biomarkers and the effectiveness of specific diagnostic techniques to examine how these studies interrelate with the broader knowledge network on glioma, which increased the number of significant articles. In contrast, the results we obtained by applying KRA suggest that only a small number of cited articles play a key role in connecting different research paradigms, as is the case with the integration of advanced imaging techniques with molecular methods for more accurate classification, seeking to show that the evolution of knowledge is neither linear nor hierarchical but depends on an interconnected set of studies that, although not the most cited, serve as a bridge and facilitate the advancement and fusion of multiple disciplines. Additionally, our approach also differs from the contributions of [35, 41] and [12] in that they connect the literature on gliomas with topics generally related to aspects such as predictive models or cognitive impairment without addressing how the studies relate to disease classification or how classification criteria could be expanded through social factors.

Another aspect we seek to highlight from the network perspective applied to bibliometric data is the identification of densely connected substructures in the citation network through k-core analysis. While the systematic reviews collected in Table 1 focused mainly on obtaining traditional impact metrics, the approach implemented in our work sought to section and navigate the network to identify research communities that are not only influential due to their number of citations but also act as hubs where diverse lines of research converge. For example, the identification of substructures revealed the presence of research integrating genomic biomarkers with statistical modeling techniques, suggesting a move towards personalization and increased treatment accuracy and efficacy, which had not been sufficiently addressed in previous studies. Within the framework of these ideas, the application of k-core analysis also provided insight into the low degree of integration of social and behavioral factors. While [50] and [44] reported an initial exploration of these aspects, our results show that these works are underrepresented in key knowledge pathways. This suggests that, despite recognizing their importance, systematic integration of these factors into glioma research has not yet been achieved.

Our study found that the social factors incorporated into the studies that make up the network under study are those that affect quality of life, such as depression and cognitive function. We also found that age, gender, and geographic region are often used as composite indices made up of income, poverty, and education as proxies to indicate that socioeconomic status can predict disease incidence and survival rates. Other social factors that we identified and that intersect with molecular classification are the type of social coverage of the patient and the type of hospital infrastructure to which they have access as determinants of access to testing and treatment. In this regard, the work of [31] reinforces the idea that access to treatment, as well as hospital costs, are social determinants with a classificatory impact. The work of [20] recognizes ethnicity as a social dimension of disease that modulates the distribution of glioblastoma subtypes as well as clinical decisions for classification. However, to gain a more comprehensive understanding of how social factors influence treatment and disease staging, it is recommended to consider factors such as indicators of socioeconomic disadvantage at the neighborhood level, which incorporate income, education, employment, and environmental conditions. According to [97], the above is associated with incidence and survival in gliomas. As these are open and georeferenced metrics, they would strengthen their deployment and the reproducibility of records. In other words, co-stratification coupled with the WHO molecular class would allow for the a priori identification of patients at risk of diagnostic delays, under-resourcing, or treatment discontinuity and activate specific referral and support circuits. Another indicator that is not included in current classification frameworks is financial access, which adds sociocultural aspects that serve as bridges or gateways to the therapeutic circuit, such as health literacy. According to [21], patients with limited language proficiency face greater barriers to accessing cancer trials and increase the gap in the use of telemedicine, so measuring this could add actionable information to ensure that molecular stratification translates into informed decisions and adequate adherence. Another factor underrepresented in the articles reviewed is environmental and support factors, i.e., housing stability, food security, and access to informal support networks.

The contribution of our work lies in the construction of an integrative framework for exploring research on glioma classification that explicitly incorporates structural data derived from network analysis to capture the central evolutionary trajectory of the research area of interest. It also provides a robust alternative for questioning the body of knowledge in a network. For example, we explicitly question the representation of social determinants considered in classification processes. Our approach differs from conventional bibliometric reviews by integrating three techniques from graph theory and, unlike studies that report aggregate counts, co-citation, or co-occurrence of terms, the approach we adopted takes into account the temporal directionality of the influence of scientific works, structures the backbone of the field and allows us to find critical paths with high betweenness that may be inadvertently overlooked or insignificant for traditional approaches or when only the citation frequency of articles is prioritized.

The decomposition of the network using k-core analysis allowed us to find and delimit dense knowledge clusters, helping to distinguish methodological peripheries with disruptive potential. It should be noted that this approach to topology not only considers the most cited articles but also detects less cited but structurally crucial bridge works, providing a more granular reading of the dynamics of consolidation and paradigm shift that escape the scope of traditional metrics such as the h-index. In this sense, we believe that the methodological implications for interdisciplinary research are direct, as the overlap of the three techniques used to study the same network allowed us to find points of convergence between fields such as genomics and epigenetics, radiomics and neuroimaging, and population epidemiology, and to locate articles that link previously fragmented communities. Therefore, this type of structural reading would provide areas of opportunity for the design and operationalization of collaborations and for incorporating different layers of variables, such as clinical and social variables, in a reproducible manner. In other words, the same workflow allows for expansion into multimodal networks where imaging, genetics, and demography intersect, maintaining temporal consistency and the traceability of theoretical-methodological concordances. Subsequently, the methodological proposal allows us to move from the descriptive level and become a tool that enables professionals and practitioners to align laboratory, clinical, and public health agendas.

## CONCLUSIONS

We consider this article different from other systematic literature review papers on gliomas because it utilized a robust network analysis approach. Previous systematic reviews tend to focus on identifying and evaluating studies on conceptual structure (word network) and prominent authors and institutions without delving into how these elements interact with social and quality-of-life factors.

Based on the citation network analysis, we answer the questions posed at the end of the literature review. What disciplinary approaches influence the classification of gliomas? A convergence of multiple disciplinary approaches has influenced the classification of gliomas. A review of the articles indicates that the disciplines most involved are neuropathology, genetics, epigenetics, bioinformatics, and clinical oncology. Historically, histopathology is considered the most relevant as it began with studies of cellular morphology and mitotic characteristics. However, with advances in molecular biology and genomics, the disciplinary focus has shifted to integrating genetic and epigenetic data. Genetics and epigenetics have been pivotal in identifying specific mutations, improving diagnostic accuracy, and providing crucial prognostic information. Bioinformatics has become critical for managing and analyzing large volumes of genomic and epigenomic data, enabling the identification of new subtypes of gliomas based on DNA methylation profiles. The interaction of these disciplines has led to a new stage of glioma classification that is much more precise and personalized, aligning with the principles of precision medicine in oncology.

What factors have been considered to expand this classification? The review of the articles identified by the network analysis allows us to say that the broadening of the classification of gliomas has been driven by critical factors such as the need to improve diagnostic accuracy, prognostic ability, and treatment personalization. Integrating molecular biomarkers can be considered a critical factor that allows a more apparent distinction between different gliomas and helps identify subgroups of patients who may benefit from specific treatments. Another critical factor in broadening the classification of gliomas is DNA methylation profiles, which enrich the classification by epigenetic patterns and are helpful in cases where histopathological and genetic data are insufficient for a definitive diagnosis. Other factors that have served to strengthen the classification of these diseases are factors such as tumor heterogeneity, clonal evolution, and differences in the tumor microenvironment, which influence response to treatment and disease progression; this, in turn, has led to a move towards a more complex classification for more informed decision-making.

What social risk factors are associated with distinct glioma phenotypes? Although social risk factors are not explicitly mentioned in contextual terms in the studies reviewed, network analysis of the glioma literature suggests that factors affecting quality of life, such as depression, cognitive function, and other brain tumor-related symptoms, are intertwined and require a multidimensional approach for a deeper understanding of how social factors may influence disease management and staging. Specifically, the work of Coomans and Röttgering addressed the relationship between symptoms and quality of life in patients with diffuse gliomas.

There are several limitations of our work which we consider relevant to note to narrow and contextualize the scope of future work. First, we recognize that, at the time of completion, other articles may have been published that have not been included. Second, the data analyzed exclusively come from the Web of Science Core Collection, focusing on articles written in English from journals classified as Q1 or Q2. Although this aligns with our interest in constructing the paradigmatic core of the topic and taking advantage of the degree of metadata standardization, it may lead to a tendency to use high-impact journals with an Anglo-Saxon tradition, potentially underrepresenting publications in regional journals not indexed in the chosen database. Along these lines, it should be noted that we excluded gray literature, i.e., the construction of the networks did not consider technical reports, conference proceedings, or guidelines, which means that although the networks focused on formally published literature, they have limitations in capturing the practical and organizational dimension of emerging classification approaches. Therefore, incorporating additional databases could further expand the citation network. Thirdly, despite modeling the temporal distribution of the references cited and mitigating the effects of recency, the study remains subject to age biases because, based on the medical practice of one of the authors, it takes as a starting point the classification reports proposed by the WHO. Additionally, more recent works have less time to accumulate citations, while older studies may be underrepresented due to obsolescence. Subsequently, the subgraphs presented as the main path, key route, and k-core reflect behavior documented in the literature, which has reached a certain level of citation maturity. In this regard, future studies could integrate our approach with dynamic analysis or alternative indicators to characterize emerging contributions further.

We also consider it pertinent to add that our perspective emphasizes structure through the use of directed graphs, represented by MPA, KRA, and k-core decomposition, because they allow us to preserve the temporal directionality of citations and, at the same time, analyze the propagation of classification frameworks throughout the literature. However, they were not used to analyze the full text of each article. Thus, the link between the identified structures and conceptual themes such as methylation, HDI, or imaging biomarkers was established through manual content analysis of key papers rather than through automatic topic modeling or semantic representation techniques. Despite these limitations, we believe it may be of interest to academics, as it offers an avenue for discussion to design future studies exploring the interactions among multiple biological and social variables and their impact on clinical outcomes, thereby providing a more solid basis for innovation in the classification and treatment of gliomas. From a practical perspective for medical professionals, the approach presented may offer a way to improve the assessment and treatment of patients with gliomas, taking into account social risk factors such as the patient’s environment and support network, as well as traditional clinical symptoms. Neuro-oncology specialists can design more comprehensive treatment protocols and provide personalized care to glioma patients.

## Appendix A

List of steps performed in Pajek for citation network analysis:

—————— Importing the .net file: ——————

1. Open cite.net file.

2. Click Info button for exploration [0 in the dialog box].

3. Click on Network > Create network > Transform > Remove > Loops.

Simplifying the grap.

4. Now, remove multiple lines: Network > Create network > Transform > Remove > Multiple lines > Min value.

5. Change the label to: GliomaCite_Simp

6. Export or save the network

—————— Degree exploration: ——————

Indegree

7. Network > Create vector > Centrality > Degree > Input

8. File > Vector > Change label →Saved as: 1Can_Indeg.vec

Outdegree

9. Network > Create vector > Centrality > Degree > Output

10. File > Vector > Change label →Saved as: 1Can_Outdeg.vec

Alldegree

11. Network > Create vector > Centrality > Degree > All

12. File > Vector > Change label→Saved as: 1Can_Alldeg.vec

—————— Product type: ——————

Read the simplified network

13. Read DC.clu

14. Partition > Binarize Partition [1]

15. File > Partition > Change Label →[base]

16. Partition > Copy to Vector

17. Network > Create New Network > Transform >Transpose 1-mode

18. Operations > Network + Vector > Network * Vector [1]

19. Vector > Make Partition > Copy to Partition by Truncating

20. Partition > Binarize Partition [1-*]

21. File > Partition > Change Label →[base neighbors]

22. Select DC partition

23. Partition > Binarize Partition [0]

24. File > Partition > Change Label →[sources]

25. Select base neighbors as SECOND PARTITION

26. Partitions > Min (First, Second)

27. File > Partition > Change Label →[basic sources]

28. Partition > Binarize Partition [0] →Change label to complement

29. Select sources as SECOND PARTITION

30. Partitions > Min (First, Second)

31. File > Partition > Change Label →[secondary sources]

32. Select DC partition

33. Partition > Binarize Partition [2]

34. File > Partition > Change Label →[users]

35. Save partitions base, users, basic, and secondary to files.

—————— Boundary problem: ——————

36. Select the simplified network

37. Network > Create Partition > Degree > Input

38. Partition > Binarize Partition [2-*] (k1=1)

39. Select basic sources as Second Partition

40. Partitions > Min (First, Second)

41. File > Partition > Change Label →[basic and (indeg > 1)]

42. Select indegree partition

43. Partition > Binarize Partition [4-*] (k2=3)

44. Select secondary sources as Second Partition

45. Partitions > Min (First, Second)

46. File > Partition > Change Label →[secondary and (indeg > 3)]

47. Select DC partition

48. Partition > Binarize Partition [1-*]

49. Select secondary and (indeg > 3) as Second Partition

50. Partitions > Max (First, Second)

51. Select basic and (indeg > 1) as Second Partition

52. Partitions > Max (First, Second)

53. File > Partition > Change Label →[boundary]

54. Operations > Network + Partition > Extract Subnetwork [1]

—————— Acyclic graph: ——————

55. Select the CiteB or CancerCiteB network

56. Network > Create Partition > Components > Strong [2]

57. Operations > Network + Partition > Shrink Network [1 0]

58. Network > Create New Network > Transform > Line Values > Set All Line Values to 1 [No]

59. Network > Create New Network > Transform > Remove > Loops

60. Network > Acyclic Network > Create weighted Network + Vector > Transversal Weights > Search Path Count

—————— Suggested by the reviewer: ——————

*. Network > Acyclic Network > Create weighted Network + Vector > Transversal Weights > Search Path Link Count (SPLC).

*. Network > Acyclic Network > Create weighted Network + Vector > Transversal Weights > Search Path Node Pair (SPNP)

In this case, the network, partition, and vector were saved as 2CANCER_CITE_SPC

61. File > Network > Change label > 2CANCER_CITE_SPC

Save as: 2CANCER_CITE_SPC

This is the network that will be used for working and reporting results.

—————— Main Path Extraction: ——————

62. Network > Acyclic Network > Create SubNetwork > Main Path > Global Search > Standard

63. Network > Create Partition > Degree > All

64. Network > Create Vector > Centrality > Degree > All

65. File > Network > Change Label →[2CANCER_MainPath]

—————— Key Route Extraction: ——————

66. Network > Acyclic Network > Create SubNetwork > Main Path > Global Search > Key Routes [1-10]

67. Network > Create Partition > Degree > All

68. Network > Create Vector > Centrality > Degree > All

69. File > Network > Change Label →[2CANCER_KeyRoute]
